# Relationships between hydration biomarkers and total fluid intake in pregnant and lactating women

**DOI:** 10.1007/s00394-016-1256-3

**Published:** 2016-08-12

**Authors:** Amy L. McKenzie, Erica T. Perrier, Isabelle Guelinckx, Stavros A. Kavouras, Giselle Aerni, Elaine C. Lee, Jeff S. Volek, Carl M. Maresh, Lawrence E. Armstrong

**Affiliations:** 1 Department of Kinesiology, Human Performance Laboratory, University of Connecticut, 2095 Hillside Road, Storrs, CT 06269-1110 USA; 2 Danone Research, RD 128, 91767 Palaiseau, France; 3 Human Performance Laboratory, Department of Health, Human Performance and Recreation, University of Arkansas, 155 Stadium Drive, HPER 321, Fayetteville, AR 72701 USA; 4 University of Connecticut Health, Family Medicine, 263 Farmington Ave, Farmington, CT 06030 USA; 5 Department of Human Sciences, Ohio State University, PAES Building, 305 W. 17th Ave, Columbus, OH 43210 USA

**Keywords:** Hydration status, Urine biomarkers, Blood biomarkers, Fluid intake adequacy, Gestation, Breast-feeding

## Abstract

**Introduction:**

Previous research established significant relationships between total fluid intake (TFI) and urinary biomarkers of the hydration process in free-living males and females; however, the nature of this relationship is not known for pregnant (PREG) and lactating (LACT) women.

**Purpose:**

To determine the relationship between urinary and hematological hydration biomarkers with TFI in PREG and LACT.

**Methods:**

Eighteen PREG/LACT (age: 31 ± 3 years, pre-pregnancy BMI: 24.26 ± 5.85 kg m^−2^) collected 24-h urine samples, recorded TFI, and provided a blood sample at 5 time points (15 ± 2, 26 ± 1, 37 ± 1 weeks gestation, 3 ± 1 and 9 ± 1 weeks postpartum during lactation); 18 pair-matched non-pregnant (NP), non-lactating (NL) women (age: 29 ± 4 years, BMI: 24.1 ± 3.7 kg m^−2^) provided samples at similar time intervals. Twenty-four-hour urine volume (*U*_VOL_), osmolality (*U*_OSM_), specific gravity (*U*_SG_), and color (*U*_COL_) were measured. Hematocrit, serum osmolality (*S*_OSM_), and serum total protein (*S*_TP_) were measured in blood.

**Results:**

Significant relationships were present between TFI and urinary biomarkers in all women (*P* < 0.004); these relationships were not different between PREG and NP, and LACT and NL, except *U*_VOL_ in PREG (*P* = 0.0017). No significant relationships between TFI and hematological biomarkers existed (*P* > 0.05).

**Conclusion:**

Urinary biomarkers of hydration, but not hematological biomarkers, have a strong relationship with TFI in PREG, LACT, NP, and NL women. These data suggest that urinary biomarkers of hydration reflect TFI during pregnancy and breast-feeding.

## Introduction

As a major constituent of the human body and involved in many metabolic processes, water is an essential nutrient. Low water intake or low urine volume are associated with negative health outcomes such as increased risk of chronic kidney disease [1], rate of decline in kidney function [2], development of hyperglycemia [3], and constipation [4]. Further, recommendations for the treatment or prevention of constipation [5, 6], urolithiasis [7], and nephrolithiasis [8] include maintaining an adequate water intake, urine volume, or urine specific gravity to avoid complications. Maintenance of an appropriate hydration process—that is, a high volume of daily body water turnover (high intake, high output)—can be assessed through the measurement of urine concentration and volume [9]. In particular, urine concentration is of interest as a marker of appropriate intake as it takes into account all forms of water intake from foods and fluids, dietary solute load, and insensible and sweat losses. Urine osmolality as high as 830 mOsm kg^−1^ has been proposed as the upper limit of euhydration [10] and verified statistically in the case of acute, active dehydration [11]. However, maintaining a daily, lower 24-h urine osmolality of 500 mOsm kg^−1^ has been proposed as ‘desirable’ at the population level [12] and suggested as a target for optimal intake [13] for the reduction in disease risk. Today, the relationship between total water intake and urinary hydration biomarkers in healthy adults is well described [14–17]. Higher urine volumes are generally associated with higher levels of water or fluid intake, and more concentrated urine (higher osmolality, specific gravity and color) are associated with lower levels of water intake; these markers track closely with total water (or fluid) intake in many populations, including adult men and adult women who are not pregnant or breast-feeding.

Pregnancy and lactation, however, present exceptional challenges to water homeostasis. In pregnancy, total body water increases, plasma volume expands, and water is continuously being exchanged between the developing fetus and its environment [18]. In addition, plasma osmolality decreases and the osmotic threshold for vasopressin release is reset [19]. Postpartum, breast-feeding women are challenged by additional water loss through breast milk production averaging 700 ml d^−1^ [20]. While the effect of maternal hydration on pregnancy outcomes is unclear, evidence suggests that increasing water intake in pregnant women can acutely increase the amniotic fluid index (AFI), which estimates amniotic fluid volume and serves as an index of fetal well-being [21].

Given these changes to body water regulation and homeostasis, the relationship between fluid intake and urinary hydration biomarkers may be different during pregnancy and lactation compared to healthy, non-pregnant adults. Previous literature has tracked hydration biomarkers throughout gestation and during the postpartum period [22], but never in relation to total fluid intake. In non-pregnant, non-lactating women, previous literature has established positive relationships between total fluid intake and 24-h urine volume, but inverse relationships between total fluid intake and 24-h urine osmolality, specific gravity, and color; no significant relationships between total fluid intake and hematological biomarkers in this population have been demonstrated [23]. Thus, the purpose of this investigation was to determine whether significant relationships existed between total fluid intake and common urinary and hematological hydration biomarkers during pregnancy, while breast-feeding, and in non-pregnant, non-lactating pair-matched controls. Further, if a significant relationship between total fluid intake and a hydration biomarker was present, this investigation sought to determine whether there was a difference in this relationship between pregnant and non-pregnant women, and between lactating and non-lactating women. We also evaluated whether the relationship between total fluid intake and the biomarker changed over time within each group. We hypothesized that the same relationships between total fluid intake and urinary biomarkers of hydration would be present in pregnant and lactating women, and that there would be no difference in the relationship between control and pregnant and/or lactating women, and no difference in the relationship over time. Further, we hypothesized that no significant relationships would exist between total fluid intake and hematological biomarkers of hydration.

## Participants and methods

### Participants

Twenty pregnant women and eighteen non-pregnant, non-lactating control women volunteered to participate in this observational research study. Two pregnant women were excluded from data analysis due to incomplete data or development of a gestational condition (e.g., gestational diabetes, preeclampsia) that could alter fluid balance. All pregnant women enrolled in the study by 16 weeks of gestation and had singleton pregnancies. Participants were excluded for tobacco use, participation in strenuous exercise (≥7 h per week), or presence of a health condition (e.g., diabetes, kidney dysfunction or disease, urinary tract infection, electrolyte abnormality, etc.) or prescription of a medication (e.g., diuretics) that could alter fluid balance. The university institutional review board approved this investigation, and all participants provided written informed consent. All study procedures were performed in accordance with ethical standards specified by the Declaration of Helsinki.

### Study design

Data were collected from pregnant and subsequently lactating participants at five time points: (1) the end of the first (15 ± 2 weeks gestation) (2) second (26 ± 1 weeks gestation), and (3) third trimesters (37 ± 1 weeks gestation) as well as at (4) 3 ± 1 weeks and (5) 9 ± 1 weeks postpartum. Control women were matched to pregnant participants on the basis of age, height, and weight; these participants visited the laboratory at similar time intervals to the pregnant and lactating women and only during the early follicular phase of the menstrual cycle. This was done to limit effects of exogenous estrogen on fluid balance [24] and to ensure each visit occurred at the same point in their cycle. All control women were taking a combination drug oral contraceptive.

Participants reported age, pre-pregnancy body mass, and parity. Researchers instructed participants to eat and drink according to their normal habits throughout the investigation and also instructed participants how to maintain an accurate fluid intake record. Participants recorded the fluid they consumed 1 day before each visit to allow calculation of 24-h total fluid intake (TFI; water consumed from drinking water and other beverages). Total fluid intake was chosen as the variable of interest given its practicality, ease of measurement by women in their daily lives, and that it generally accounts for 80 % of total water intake (i.e., water from both foods and fluids), which requires dietary analysis. During this time, participants also collected their urine for 24 h using the following procedure. On the morning before each laboratory visit, participants awoke, voided, and discarded this first morning void. All subsequent voids produced throughout the day and overnight were collected in a single urine collection container. On the morning of the study visit, participants awoke, voided, and included this first morning void to complete a full 24-h collection. Participants then returned the urine collection container to investigators at the beginning of each laboratory visit; no participant reported missed voids.

Each laboratory visit occurred during the morning and at a similar time across all five visits. Participants were fasted for at least 4 h before each visit but were allowed to consume water ad libitum. At the beginning of each visit, researchers recovered the 24-h urine collection, reviewed each participant’s fluid intake record, checked any entries that were unclear, and probed for possible omissions. Participants measured fluid volumes with wet measuring cups when the exact volume was not available on a container and recorded their fluid logs in real time throughout the 24 h. Body mass was recorded to the nearest 0.01 kg (Health O Meter, Model 349KLX, Alsip, IL), and height was measured on the first visit via stadiometer. Body mass index was calculated as body mass (kg) divided by height^2^ (m^2^). Venous blood was drawn into Vacutainer^®^ (BD Biosciences, Franklin Lakes, NJ) tubes without additive (centrifuged for serum) and with K_2_EDTA (for whole blood). Osmolality (*S*_OSM_) via freezing point depression and total protein (*S*_TP_) via refractometer were measured in the serum. Hematocrit (Covidien, Microhematocrit Tube Reader, Minneapolis, MN) was measured in K_2_EDTA-treated whole blood.

The 24-h urine sample was assessed for volume (*U*_VOL_) gravimetrically (Ohaus Corporation, Ranger 3000, Parsippany, NJ), osmolality (*U*_OSM_) by freezing point depression (Advanced Instruments Inc., Model 3320, Norwood, MI), and specific gravity (*U*_SG_) by manual refractometer (Reichert Technologies, Model TS-400, Depew NY). Urine color (*U*_COL_) of 24-h samples was observed by a single investigator in a well-lit room by observing urine in a clear container against a white background, adjacent to a previously published 8-category urine color chart [25]. The investigator recorded the number of the color that best matched the sample; the darker of two colors was recorded if the sample color was determined to fall between two colors on the chart for consistency of technique. At visit 4 (3 ± 1 weeks postpartum), lactating women also recorded 24-h breast milk volume (*M*_VOL_), measured directly at time of expression if pumped, or estimated by test-weighing the infant to the nearest 10 g (Newline Digital Weight Track, Model SHAEBSA-20, Edgewood, NY) at each feeding as previously described [20], during the same 24 h in which they collected urine and recorded their fluid consumption. Research staff trained the mothers on how to use the infant scale and on the procedure for test-weighing infants before and after feedings.

### Statistical methods

Data analyses were performed with SAS (version 9.4, SAS Institute Inc., Cary NC). Differences in anthropometric and demographic characteristics were assessed at the first visit during pregnancy (visit 1) and during lactation (visit 4) with independent samples t tests. We utilized a repeated measures analysis of covariance with three covariates (TFI, group, and time). Individual intercepts and slopes of TFI were fitted for each combination of group and time. F tests were performed to determine whether there was a significant relationship (nonzero slope) between TFI and each hydration biomarker. If a significant relationship (nonzero slope) was present, homogeneity of regression coefficients via *F* tests determined whether the relationships (slopes) were similar between groups and time. For group comparisons, pregnant (PREG; end of the first, second, and third trimesters) were compared to non-pregnant (NP; visits 1–3) women, and lactating (LACT; 3 ± 1 and 9 ± 1 weeks postpartum) were compared to non-lactating (NL; visits 4–5) women to test for between-group differences, within each visit. Time comparisons of slopes were performed separately for pregnancy (visits 1–3) and lactation (visits 4–5), within each group (PREG, LACT, NP, NL). If significant differences between groups or time were noted, post hoc comparisons with a Bonferroni adjustment were utilized to determine where differences in the relationship existed. We utilized a simple linear regression to determine whether a relationship between TFI and *M*_VOL_ was present in LACT at 3 ± 1 weeks postpartum.

## Results

### Participant characteristics and total fluid intake

**Fig. 1:**
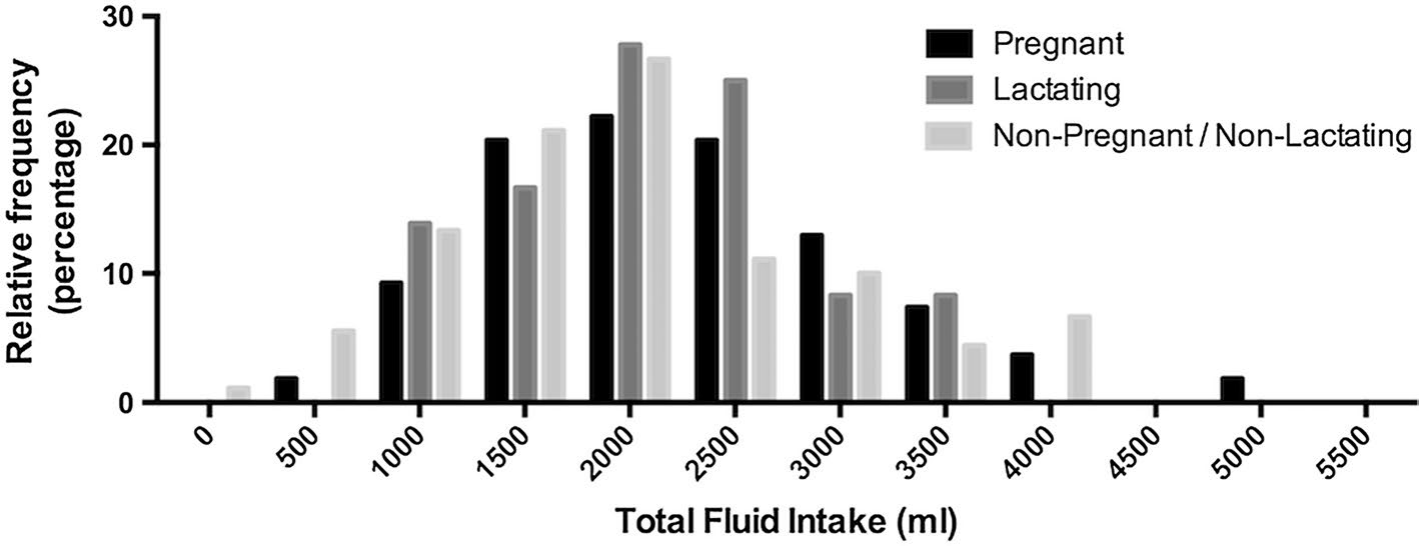
Relative frequency of total fluid intakes within each group. Total fluid intake values on the *x*-axis reflect the center of the grouping, which spans 500 ml

**Fig. 2:**
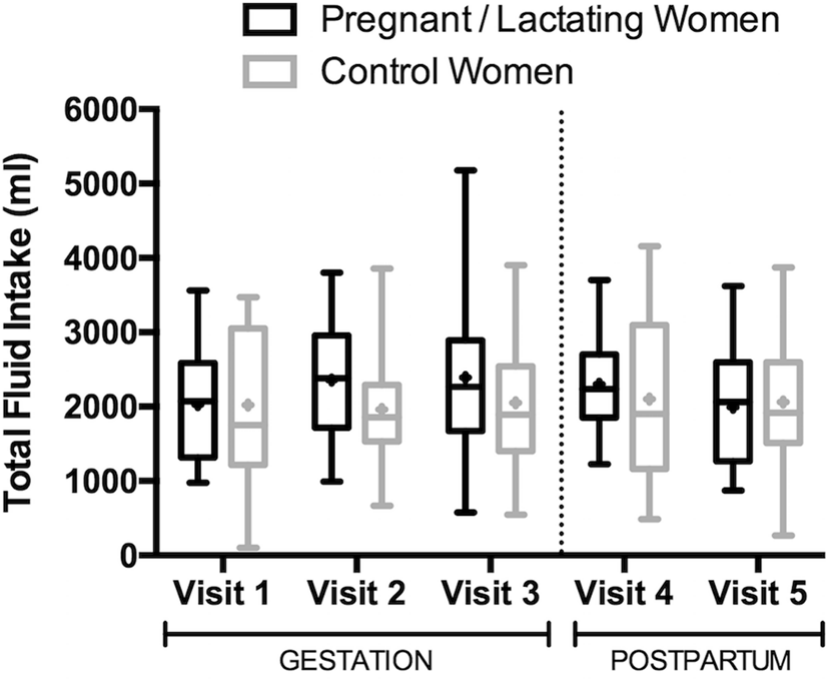
Total fluid intake by group across visits. *Boxes* depict the 25th percentile, median, and 75th percentile of distribution. Whiskers reach the minimum and maximum. Mean values are represented by + symbols

Demographic characteristics and anthropometrics between groups were not statistically different at the end of the first trimester or at 3 ± 1 weeks postpartum (Table 1; *P* > 0.05). Body mass of NP and NL women remained constant over the course of the study; the group mean fluctuated by <1 kg and <1 % of body mass. Pregnant women gained 5.97 ± 2.47 kg and 10.52 ± 3.74 kg at the end of the second and third trimesters, respectively, compared to the end of the first trimester. At 3 ± 1 and 9 ± 1 weeks postpartum, lactating women weighed 2.48 ± 3.95 and 1.89 ± 3.69 kg more, respectively, than at the end of the first trimester. Total fluid intake consumed at all visits spanned a wide range (103–5175 ml; Fig. 1). Daily TFI during pregnancy and lactation was 2259 ± 878 ml (range: 577–5175 ml) and 2148 ± 749 ml (range: 872–3704 ml), respectively; non-pregnant and non-lactating women consumed a TFI of 2041 ± 916.7 ml (range: 103–4155 ml; Fig. 2).

**Table 1:** Summary of participant anthropometrics and demographics

	Pregnant	Non-pregnant	*t*	*P*
Visit 1 (end of the first trimester, 15 ± 2 weeks gestation)				
*n*	18	18		
Parity	1 ± 1	0 ± 0		
Age (years)	31 ± 3	29 ± 4	1.382	0.176
Height (cm)	165.5 ± 6.7	163.7 ± 8.3	0.752	0.458
Body mass (kg)	69.58 ± 18.57	64.83 ± 13.84	0.870	0.390
Self-reported pre-pregnancy body mass (kg)	66.89 ± 19.24	64.70 ± 14.28	0.368	0.715
Pre-pregnancy BMI (kg m^−2^)	24.26 ± 5.85	24.05 ± 3.70	0.134	0.895

	Lactating	Non-lactating	*t*	*P*
Visit 4 (3 ± 1 weeks postpartum)				
*n*	18	18		
Age (years)	31 ± 3	30 ± 4	1.211	0.234
Body mass (kg)	72.06 ± 17.26	64.83 ± 13.51	1.399	0.171
BMI (kg m^−2^)	26.14 ± 5.21	24.06 ± 3.69	1.384	0.176

### Relationship between TFI and hydration biomarkers during pregnancy

**Fig. 3:**
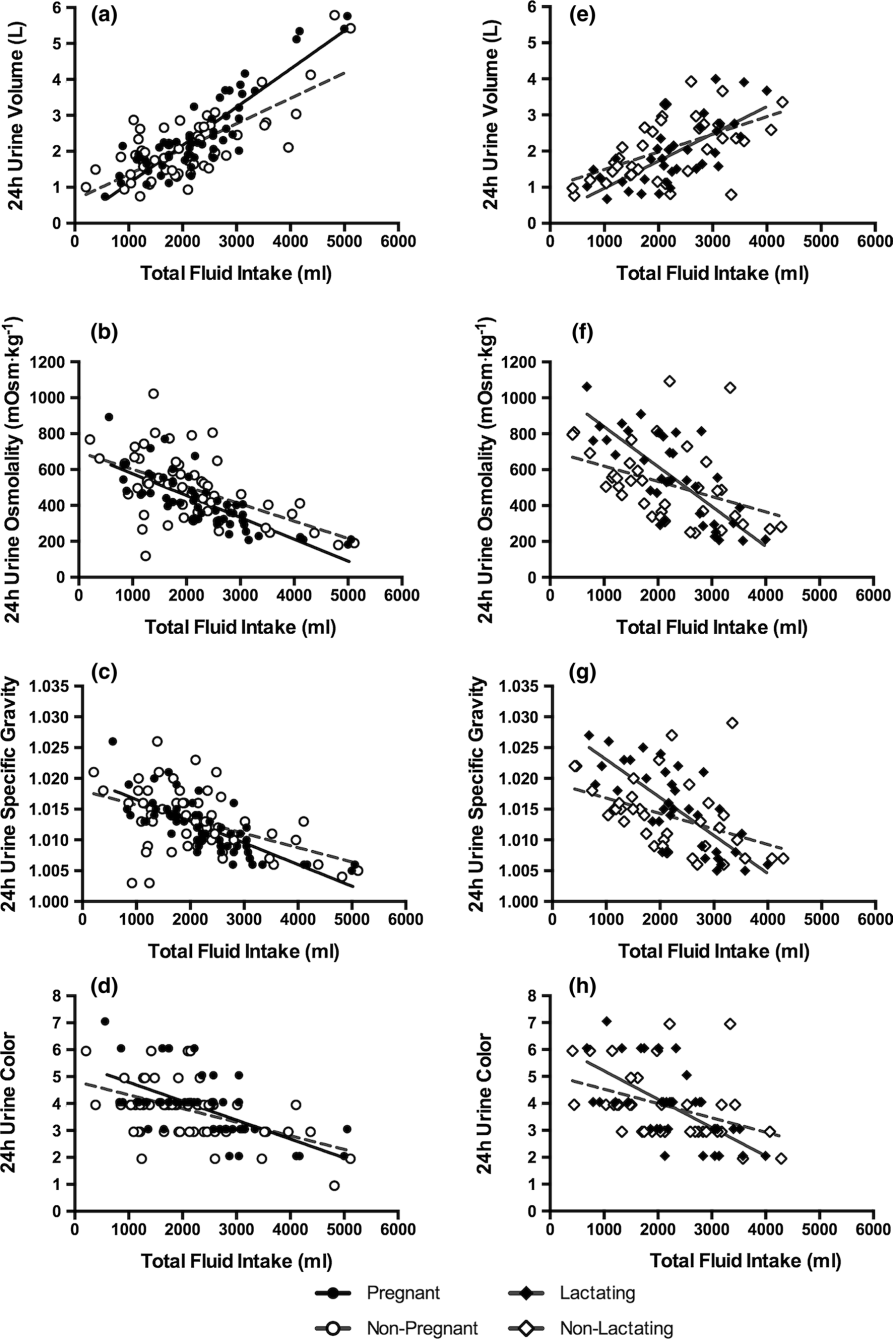
Relationships between TFI and urinary hydration biomarkers. A significant relationship existed between TFI and all urinary hydration biomarkers in all groups. *Solid lines* reflect the trend line for the relationship in PREG–LACT; *dotted lines* reflect the trend line for the relationship in NP–NL (all visits combined). The slope of the regression line in PREG was significantly different from NP for the relationship between 24-h *U*_VOL_ and TFI within visit (*F* = 4.11, *P* = 0.0047, see **a**), with the difference occurring at visit 3 (*t* = 3.38, *P* = 0.0017). The relationship between TFI and 24-h *U*_SG_ was also significantly different between PREG and NP (*F* = 2.56, *P* = 0.0441), but no post hoc differences were identified after accounting for the number of comparisons

**Fig. 4:**
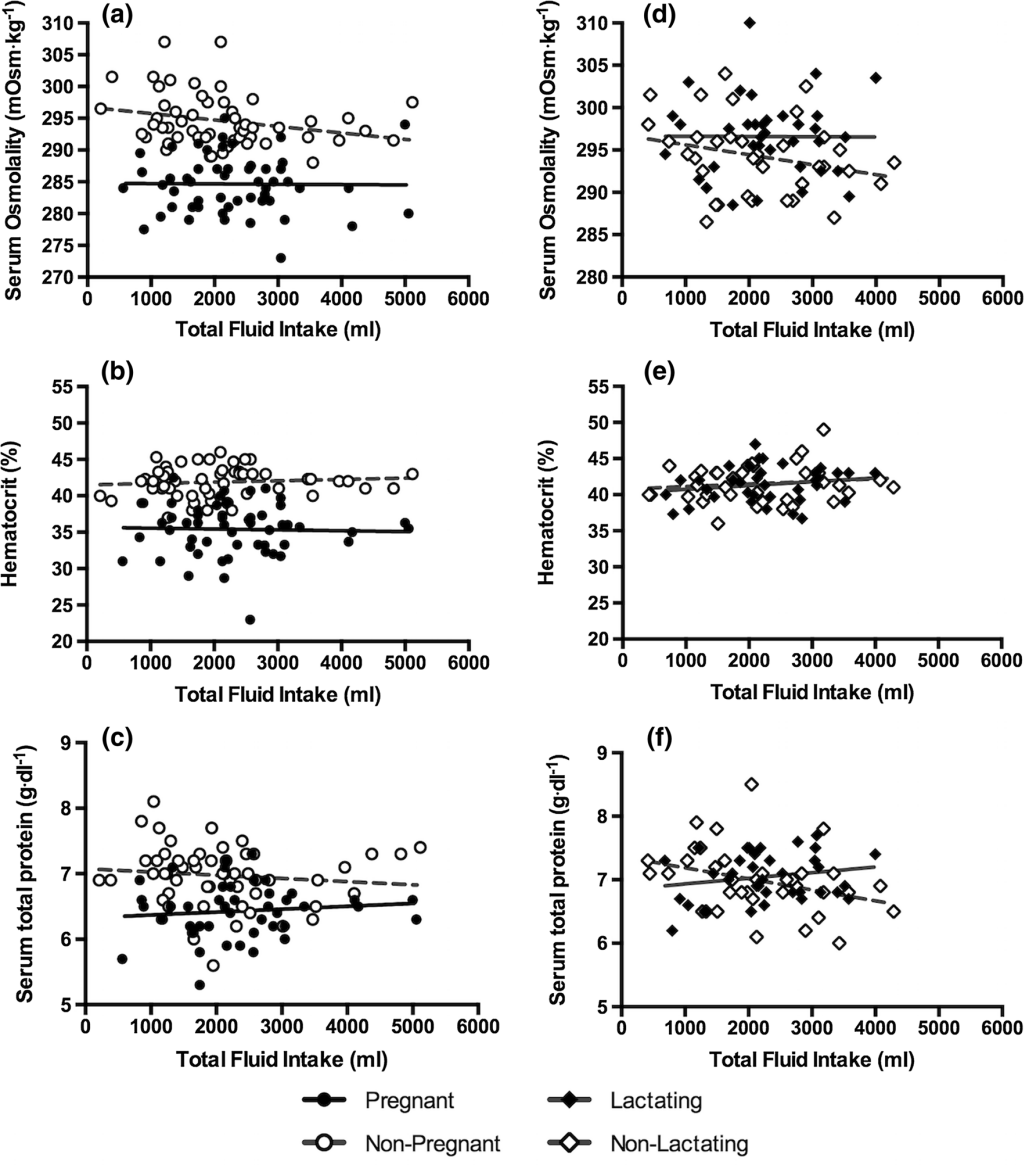
Relationships between TFI and hematological hydration biomarkers. No significant relationships between TFI and hematological biomarkers were present. *Solid lines* reflect the trend line for the relationship in PREG–LACT; *dotted lines* reflect the trend line for the relationship in NP–NL (all visits combined)

Significant relationships existed between TFI and each urinary biomarker in PREG and NP women (Table 2; Fig. 3, *P* < 0.004). These relationships were similar between PREG and NP within each visit (Table 2, Fig. 3, *P* > 0.05), except for the relationship between TFI and 24-h *U*_VOL_ (*F* = 4.11, *P* = 0.0047) and 24-h *U*_SG_ (*F* = 2.56, *P* = 0.0441). Post hoc comparisons revealed the slope of the relationship between TFI and 24-h U_VOL_ was significantly different between PREG and NP at visit 3 (*t* = 3.38, *P* = 0.0017), but no differences within a single visit were present for 24-h *U*_SG_ after adjusting for the number of comparisons. No significant relationships existed between TFI and any hematological biomarker in PREG or NP women (Table 3; Fig. 4, *P* > 0.05). However, across all time points, serum osmolality, hematocrit, and serum total protein were lower in PREG than NP.

**Table 2:** Relationship between total fluid intake and urinary hydration biomarkers and homogeneity of the regression slopes

Contrasts	24-h urine volume		24-h urine osmolality		24-h urine specific gravity		24-h urine color	
	*F* value	*P*	*F* value	*P*	*F* value	*P*	*F* value	*P*
*During pregnancy*								
Slopes equal to zero?	24.28	<0.0001	13.24	<0.0001	13.03	<0.0001	10.53	<0.0001
Slopes similar between groups within visit?	4.11	0.0047	1.93	0.1133	2.56	0.0441	2.27	0.0680
Slopes similar over time within PREG?	0.76	0.4752	0.26	0.7730	0.39	0.6832	0.56	0.5764
Slopes similar over time within NP?	6.67	0.0034	4.47	0.0185	5.59	0.0079	3.84	0.0309
*During lactation*								
Slopes equal to zero?	4.66	0.0039	6.72	0.0004	6.82	0.0004	4.66	0.0039
Slopes similar between groups within visit?	0.71	0.5535	1.26	0.3026	1.62	0.3234	0.71	0.5535
Slopes similar over time within LACT?	0.46	0.5016	0.56	0.4610	0.22	0.5666	0.3	0.5869
Slopes similar over time within NL?	0.33	0.5709	0.05	0.8279	0.37	0.9857	0.03	0.8538

**Table 3:** Relationship between total fluid intake and hematological hydration biomarkers

Contrasts	Serum osmolality		Hematocrit		Serum total protein	
	*F* value	*P*	*F* value	*P*	*F* value	*P*
*During pregnancy*						
Slopes equal to zero?	1.32	0.2737	1.19	0.3326	0.72	0.7355
*During lactation*						
Slopes equal to zero?	0.43	0.7838	0.98	0.4294	2.07	0.1055

### Relationship between TFI and hydration biomarkers during lactation

**Fig. 5:**
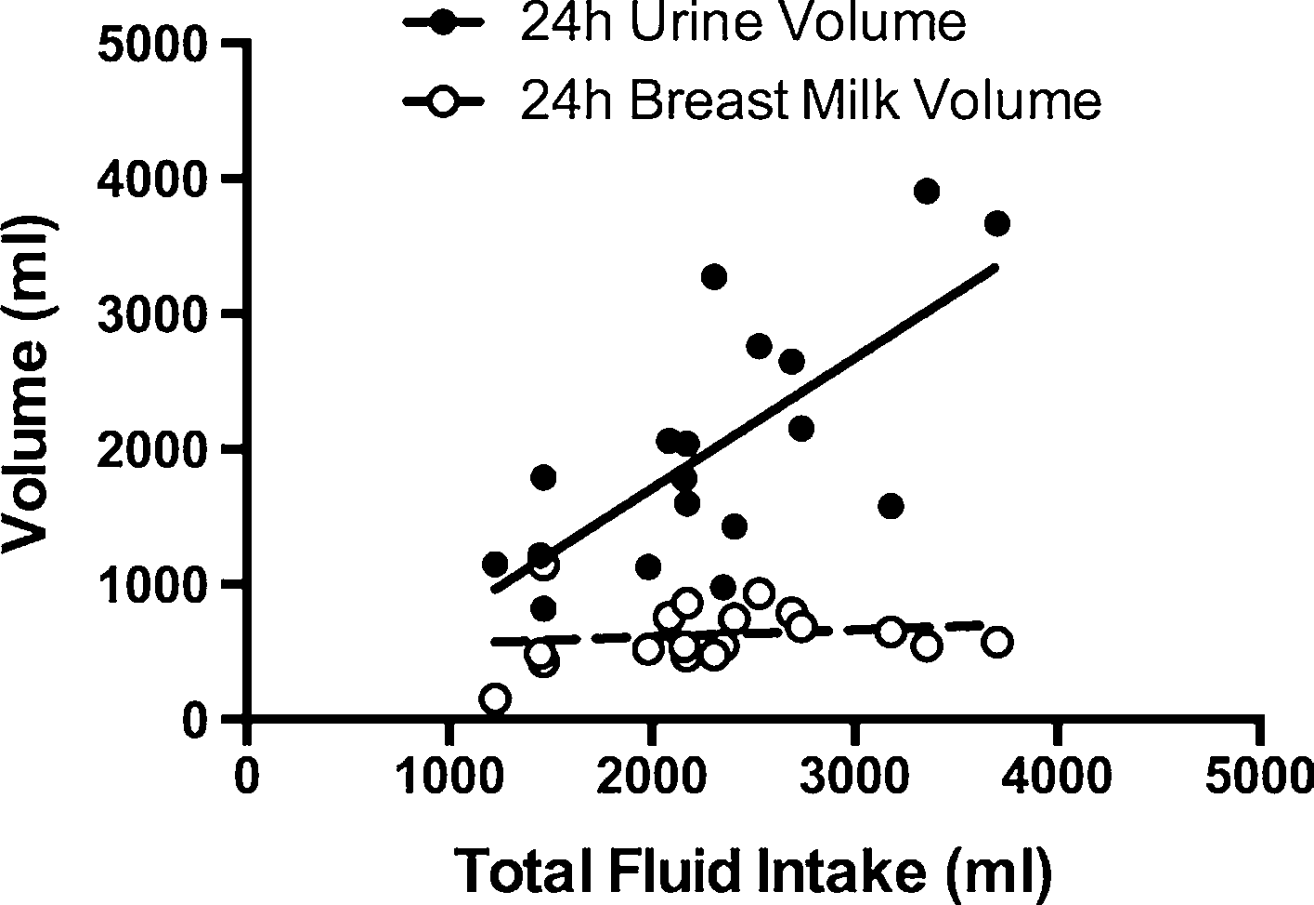
Relationship between TFI and 24-h breast milk and urine volumes in lactating women at 3 ± 1 weeks postpartum. There was a significant relationship between TFI and 24-h *U*_VOL_ in lactating women (*n* = 18, *r* ^2^ = 0.501, beta = 0.963, *F* = 16.06, *P* = 0.001) but no significant relationship between TFI and 24-h *M*_VOL_ (*n* = 18, *r* ^2^ = 0.024, beta = 0.051, *F* = 0.388, *P* = 0.542)

Significant relationships were observed between TFI and each urinary biomarker in LACT and NL women (Table 2; Fig. 3, all *P* < 0.004). The relationships between TFI and urinary biomarkers were similar between LACT and NL within each visit (Table 2; Fig. 3, *P* > 0.05). No significant relationships existed between hematological biomarkers and TFI in LACT and NL women (Table 3; Fig. 4, all *P* > 0.05). In LACT, TFI and *M*_VOL_ at 3 ± 1 weeks postpartum were not related (Fig. 5, *r* ^2^ = 0.024, *F* = 0.388, *P* = 0.542).

### Time-dependent relationship between TFI and hydration biomarkers

In PREG, LACT, and NL, the slopes of the relationship between TFI and each urinary biomarker were similar at each visit (1st = 2nd = 3rd trimester in PREG, and visit 4 = visit 5 in LACT and NL; Table 2; all *P* > 0.05). However, in NP, the slopes of the relationship between TFI and each urinary biomarker were different over time (Table 2; all *P* < 0.05). For each urinary biomarker, visit 3 was significantly different from visit 1 (*U*_VOL_: *t* = 3.65, *P* = 0.008; *U*_OSM_: *t* = −2.99, *P* = 0.0050; *U*_SG_: *t* = −3.34, *P* = 0.0019; *U*_COL_: *t* = −2.67, *P* = 0.0090); visit 2 was not significantly different from visit 1 or visit 3 (both *P* > 0.0167).

## Discussion

In this investigation, we sought to evaluate the relationship between TFI and hydration biomarkers in blood and urine of pregnant and lactating women and to determine whether these relationships were similar to those demonstrated in non-pregnant, non-lactating women. *F* tests comparing the slopes to zero revealed no significant relationships between TFI and hematological hydration biomarkers (*S*_OSM_, hematocrit or *S*_TP_) in any group of women, though hematological markers were expectedly lower in PREG across all time points. Significant linear relationships between TFI and urinary biomarkers previously found in adult men and non-pregnant women [16] also were present in pregnant and lactating women; specifically, in the current investigation, significant associations between TFI and 24-h *U*_VOL_, *U*_OSM_, *U*_SG_, and *U*_COL_ were consistently present in PREG, LACT, NP, and NL women (Fig. 2).

A statistically significant difference between PREG and NP was noted at visit 3 in the relationship between TFI and *U*_VOL_, and in NP, the relationship between TFI and all urinary biomarkers at visit 3 was significantly different from visit 1. Although the relationship was statistically different between visits 1 and 3 in NP, the direction of the relationship (i.e., the direct relationship between *U*_VOL_ and TFI and the inverse relationship between TFI and *U*_OSM_, *U*_SG_, and *U*_COL_) remained the same. Steady, rolling enrollment of NP over approximately 12 months eliminates the possibility of a seasonal effect or equipment malfunction on these differences, and the consistency with which the difference is present in all urine variables indicates a human data collection error is unlikely. It is possible that other factors (e.g., other means of fluid loss such as sweat, unreported incomplete urine collections, larger amount of water consumed from food sources than usual, etc.) altered this relationship in the NP group at this time point. Although statistical differences existed between visits 1 and 3 within non-pregnant women and between non-pregnant and pregnant women at visit 3 for urine volume, the lack of consistency over time within group and across indices between groups indicates these differences may not be clinically meaningful, and these relationships warrant further investigation with a larger sample. Despite some statistical differences noted, the consistency in the direction of the relationship between intake and output variables demonstrates that the relationships previously described between TFI and urinary biomarkers in men and non-pregnant, non-lactating women, are present and clinically similar in both pregnant and lactating women. This indicates that urinary biomarkers can be utilized as clinical indicators of TFI in this new population.

### Hematological hydration biomarkers and total fluid intake

The range in TFI observed in the present investigation is representative of the population as described in previous research [14, 23]. The absence of significant relationships between TFI and hematological biomarkers of the hydration process indicates that blood parameters are tightly regulated and defended across a range of TFI in women, including those who are pregnant or lactating (Fig. 4). Previous research demonstrated no between-group differences of *S*_OSM_ when subjects with high-volume (2.7 ± 0.4 L) and low-volume (0.74 ± 0.37 L) TFI were compared [26]. Serum osmolality also does not change when habitual high-volume drinkers restrict fluid intake or when habitual low-volume drinkers increase fluid intake [17, 27]. However, hematocrit (+1 %) and total plasma protein (+0.2 g dL^−1^) increased when high-volume drinkers decreased fluid intake, and total plasma protein differed between habitual high- and low-volume drinkers (7.0 versus 7.3 g dL^−1^, respectively) [27]. Overall, hematological biomarkers, including S_OSM_, at a single time point were not indicative of 24-h total fluid intake in pregnant, lactating, non-pregnant, or non-lactating women.

### Urinary hydration biomarkers and total fluid intake

Early research reported significant differences in 24-h *U*_VOL_ between pregnant and non-pregnant women, with pregnant women producing about 300 ml more urine per day [28]. However, recent literature has established significant relationships between 24-h urine biomarkers of hydration and TFI in adult men and women [16], indicating that this early finding could have resulted from a TFI difference between pregnant and non-pregnant women. These data demonstrate that 24-h *U*_VOL_ has a significant relationship with TFI, and the relationship between urinary hydration biomarkers and TFI remains present in pregnant and lactating women.

Given the physiological challenges to water balance and increased water needs during pregnancy and lactation, one may anticipate the relationship between intake and output to be different in this population. Statistically, the relationship between TFI and 24-h *U*_VOL_ was different between pregnant and non-pregnant women at visit 3. This may reflect the mother’s need to defend serum sodium since maternal and fetal serum sodium track closely; serum sodium is often lower during pregnancy (~138 mmol/l compared to ~142 mmol/l at 1 week postpartum [22]), and maternal hyponatremia induces fetal hyponatremia [29] as the osmotic gradient across the placenta causes increased maternal-to-fetal water movement and acute fetal diuresis [30]. To avoid maternal and fetal hyponatremia, pregnant women may quickly regulate excess fluid via a suppression of vasopressin release, resulting in higher urine volumes compared to non-pregnant women at higher TFI. In animal models, hyperhydrated pregnant goats presented with a higher urine volume compared to non-pregnant goats, while fluid-deprived pregnant goats produced less urine compared to non-pregnant goats [31], demonstrating an increased sensitivity of hydration biomarkers to total fluid. However, one limitation in the present data should be considered. Because the slope of the relationship between TFI and 24-h *U*_VOL_ was not similar between visits 1 and 3 in NP, it is possible that factors outside of those evaluated in the present investigation may have altered the relationship between TFI and urinary biomarkers in the NP group as discussed previously, and this may contribute to the between-group differences noted. Although some statistical differences existed between visits within non-pregnant women and between groups in non-pregnant and pregnant women, these statistical findings may not be clinically relevant, warranting further investigation of these relationships.

In lactating women, a significant relationship between TFI and each urinary hydration biomarker was present. No statistical differences between groups were noted within each visit postpartum. Further, lactating women defended breast milk volume across a wide range of TFI as shown in Fig. 5 of the present investigation and in past literature [32–36]. However, the defense of breast milk volume and concentration does not come without cost to the mother when TFI is low. Lactating women who consumed lower TFI presented with higher urine concentrations than non-lactating women consuming the same amount of total fluid (Fig. 3f). Given the recommendations to maintain lower urine concentrations for the prevention of negative health consequences [5, 7, 8], lactating women should be aware of the higher urine concentrations produced when their fluid intake is low. It is also interesting to note that while public health reference values for total fluid intake are higher for lactating women [12, 23], the women in this investigation did not increase fluid intake during that time. Future research might evaluate potential behavioral or physiological barriers to increased fluid intake while nursing and investigate means of eliciting behavior change in these women given their tendency toward higher urine concentrations at low fluid intakes. Educating nursing mothers on how to achieve a 24-h urine color of 3 or less may be a practical and valuable tool for clinicians in order to help these women maintain an appropriate hydration process and healthy urine concentration [37].

In conclusion, urinary biomarkers of the hydration process were related to total fluid intake in pregnant and lactating women (Fig. 3; Table 2), but hematological markers were not (Fig. 4; Table 3). These data extend previous findings that urinary hydration biomarkers were related to total fluid intake in men and non-pregnant, non-lactating women [16] to new populations: pregnant and lactating women. The strong relationship between urinary biomarkers of hydration and total fluid intake makes them useful indices of total fluid intake. Physicians and dietitians may use these data to inform clinical decisions and to advise patients or clients about fluid intake during pregnancy and lactation. Further, these findings demonstrate that urinary biomarkers may be more sensitive to differences in total fluid intake in pregnant women, highlighting the importance of mothers achieving an adequate, but not excessive, fluid intake and avoiding potential negative health consequences.
